# Advanced Home-Based Shoulder Rehabilitation: A Systematic Review of Remote Monitoring Devices and Their Therapeutic Efficacy

**DOI:** 10.3390/s24092936

**Published:** 2024-05-05

**Authors:** Martina Sassi, Mariajose Villa Corta, Matteo Giuseppe Pisani, Guido Nicodemi, Emiliano Schena, Leandro Pecchia, Umile Giuseppe Longo

**Affiliations:** 1 Department of Engineering, Università Campus Bio-Medico di Roma, Via Álvaro del Portillo, 21, 00128 Rome, Italy; martina.sassi@unicampus.it (M.S.); e.schena@unicampus.it (E.S.); leandro.pecchia@unicampus.it (L.P.); 2 Fondazione Policlinico Universitario Campus Bio-Medico di Roma, Via Álvaro del Portillo, 200, 00128 Rome, Italy; 3 Research Unit of Orthopaedic and Trauma Surgery, Department of Medicine and Surgery, Università Campus Bio-Medico di Roma, Via Alvaro del Portillo, 21, 00128 Rome, Italy; mj.villacorta.s@gmail.com (M.V.C.); mg.pisani@alcampus.it (M.G.P.); guido.nicodemi@alcampus.it (G.N.)

**Keywords:** shoulder, rotator cuff, home-based rehabilitation, exercises, monitoring devices, orthopedics

## Abstract

Shoulder pain represents the most frequently reported musculoskeletal disorder, often leading to significant functional impairment and pain, impacting quality of life. Home-based rehabilitation programs offer a more accessible and convenient solution for an effective shoulder disorder treatment, addressing logistical and financial constraints associated with traditional physiotherapy. The aim of this systematic review is to report the monitoring devices currently proposed and tested for shoulder rehabilitation in home settings. The research question was formulated using the PICO approach, and the PRISMA guidelines were applied to ensure a transparent methodology for the systematic review process. A comprehensive search of PubMed and Scopus was conducted, and the results were included from 2014 up to 2023. Three different tools (i.e., the Rob 2 version of the Cochrane risk-of-bias tool, the Joanna Briggs Institute (JBI) Critical Appraisal tool, and the ROBINS-I tool) were used to assess the risk of bias. Fifteen studies were included as they fulfilled the inclusion criteria. The results showed that wearable systems represent a promising solution as remote monitoring technologies, offering quantitative and clinically meaningful insights into the progress of individuals within a rehabilitation pathway. Recent trends indicate a growing use of low-cost, non-intrusive visual tracking devices, such as camera-based monitoring systems, within the domain of tele-rehabilitation. The integration of home-based monitoring devices alongside traditional rehabilitation methods is acquiring significant attention, offering broader access to high-quality care, and potentially reducing healthcare costs associated with in-person therapy.

## 1. Introduction

The shoulder joint is essential to activities of daily living (ADLs), facilitating fundamental needs like eating, dressing, and personal hygiene [1]. Shoulder pain represents the most frequently reported musculoskeletal disorder [2,3,4], entailing discomfort, limited range of motion (ROM), and decreased functional abilities, all of which significantly affect the quality of life [5]. Rotator cuff tendinitis, impingement syndrome, rotator cuff lesions, and adhesive capsulitis constitute the primary etiologies [1,6,7]. Despite the numerous advantages they offer, traditional rehabilitation programs have limitations for many patients, specifically in terms of accessibility, cost, and time commitment [8,9]. Recognized as an alternative or complementary approach to conventional rehabilitation programs, home-based rehabilitation empowers patients by promoting autonomy and facilitating their recovery, enabling them to regain the ability to perform ADLs [6]. The potential advantages of tele-rehabilitation systems are for both patients and healthcare organizations, including reduced travel time, cost savings, and enhanced patients’ accessibility [10]. Additionally, they can ensure continuous care, and enable health professionals to oversee the advancement of multiple patients simultaneously. The repetitive and tedious nature of prolonged at-home rehabilitation exercises can diminish patient adherence, negatively impacting functional recovery outcomes [11,12]. The success of home-based rehabilitation relies on patient engagement, adherence to the exercise regimen, and precise execution of exercises [1,13,14]. High-quality home-based rehabilitation requires technologies and methods for effective patient monitoring.

Recent advancements in remote monitoring technologies, particularly in the domain of e-health, have enabled the transition of various devices from clinical to a home setting. These devices are now designed to be more compact, smaller, ergonomic, and user-friendly, to be managed by non-health professionals as well [6]. Nowadays, different technologies have emerged to support home-based physical rehabilitation, including robotic exoskeletons, wearable systems, and contactless systems [15,16,17]. These devices facilitate remote monitoring, focusing on different primary outcomes such as assessing physical activity levels, balance and/or gait, biomechanical performance (ROM), or functional capabilities [18,19]. The integration of artificial intelligence (AI) into home-based shoulder rehabilitation systems has been transformative [5,20,21,22]. AI enhances these systems through real-time detection of compensatory movements and precise data analysis, enabling tailored treatment plans [23,24]. This advancement allows healthcare professionals to customize rehabilitation programs based on individual patient needs, revolutionizing patient care [6,25,26,27]. However, most of the systems designed for shoulder rehabilitation in home settings are typically assessed exclusively in laboratory or in-clinic environments. The execution of physiotherapy exercises may exhibit greater variability in the at-home environment, introducing additional challenges for an accurate evaluation.

With the purpose of delineating advancements and available options in this field, the aim of this systematic review is to report the monitoring devices currently proposed and tested for shoulder rehabilitation in home settings.

## 2. Materials and Methods

### 2.1. Research Question

The research question was formulated following the PICO framework: Population (P), Intervention (I), Comparator (C), and Outcome (O). In adults with shoulder injuries undergoing rehabilitation at home (P), this systematic review aims to assess whether the implementation of remote monitoring technologies (I), compared to traditional shoulder rehabilitation methods without remote monitoring or AI support (C), results in improved outcomes, including range of motion, pain reduction, and patient satisfaction (O).

### 2.2. Search Strategy

Articles were selected from two different databases, namely, PubMed and Scopus. The search strategy included free text terms and Mesh (Medical Subject Headings) terms combined with logical Boolean operators (AND, OR). In each database, the keywords and their synonyms were classified into four groups: medical application; technology and kinematic/physiological data; artificial intelligence; body segment (see Table 1). All studies published from 2014 up to 2023 were considered.

### 2.3. Eligibility Criteria

This systematic review included studies that met the following inclusion criteria: articles written in English language; investigation of shoulder rehabilitation exercises; studies focusing on the shoulder joint; availability of full text in open access.

Articles were excluded if at least one of the following criteria was met: inaccessible articles; papers published or presented at conference; reviews, books, and editorials; shoulder joint not included in the analysis; use of prosthesis, orthoses, exoskeleton, or robotic devices; patients with neurological pathologies (e.g., cerebral palsy, dystonia, hemiparesis, stroke); patients with neurodegenerative pathologies (e.g., Parkinson disease); patients with severe or moderate upper limb hemiparesis; patients who suffer from hemiplegia (complete paralysis) or hemiparesis (partial weakness) condition or paralysis; amputees’ patients; wheelchair users; studies with nonhuman subjects; studies exclusively involving healthy participants.

### 2.4. Study Selection Process

After removal of duplicates, all articles were evaluated through a first screening of title and abstract by one reviewer (M.S.). Then, the full-text evaluation of the selected papers was conducted by three independent reviewers (M.S., G.N., and M.G.P.). In cases of disagreement, the final consensus was reached after discussion with a fourth reviewer (M.V.C.).

**Figure 1:**
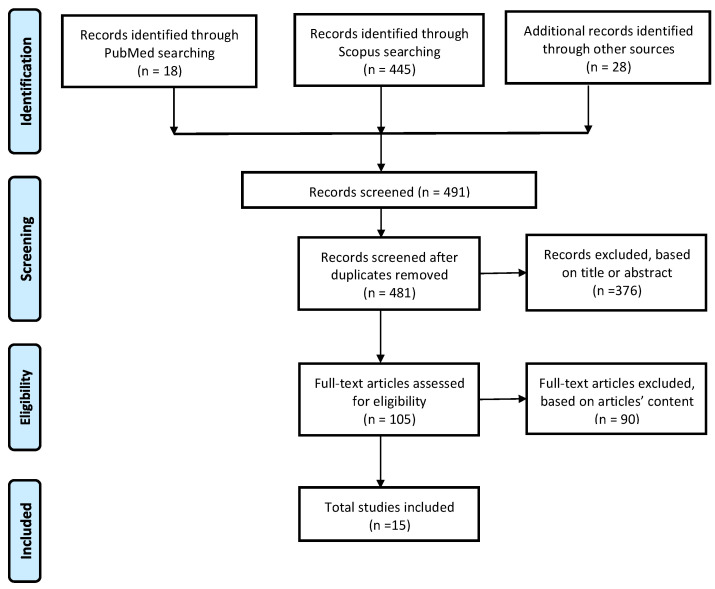
PRISMA flowchart.

A PRISMA (Preferred Reporting Items for Systematic Reviews and Meta-Analyses) flowchart was used to track the number of articles that were excluded or included at each phase. For designing the PRISMA, the guidelines of Liberati et al. [28] were followed (see Figure 1).

### 2.5. Data Items

Data extraction was executed on the 15 selected articles. General study characteristics were extracted on the basis of the following checklist: first author, year of publication; study design; level of evidence; input variable; output variable; sample size; mean age; percentage of female patients; shoulder disease; typology of the monitoring system used; number, brand, placement, and wearability of the sensors; tasks executed in the assessment protocol (number and typology); recognition of the movements; joint detection; joint angle estimation; other target variables analyzed (also physiological parameters); AI model used; system performances.

### 2.6. Data Analysis

The data from the included studies were schematized and analyzed through a combination of quantitative and qualitative approaches, providing a comprehensive overview of the home devices for shoulder rehabilitation.

### 2.7. Quantitative Synthesis

The quantitative data across the studies were analyzed to evaluate the efficacy and precision of AI and/or machine learning (ML) methods integrated within the rehabilitation devices. Performance metrics, including Area Under the ROC Curve (AUROC), accuracy, sensitivity, and specificity, were gathered for each study. Given the diversity in study designs and outcomes, a meta-analysis was not conducted. Instead, a narrative synthesis was used, where the data were described using frequencies and averages, and the efficacy patterns were summarized through descriptive statistics. The AUROC emerged as a critical measure, with values above 0.7 viewed as acceptable [21], and those surpassing 0.9 seen as exemplary in terms of the models’ discriminative capacities.

### 2.8. Qualitative Synthesis

The qualitative data were examined through thematic analysis, focusing on the objectives of the studies, the shoulder conditions treated, the rehabilitation exercises implemented, and the AI models applied.

### 2.9. Integration of Findings

The integration of qualitative themes with quantitative performance indicators highlighted an advancement in the sophistication of home rehabilitation devices. The AI/ML methods used for the recognition and correction of movement demonstrated reliable performance in controlled environments. Nevertheless, the variability in the methodological quality of the studies and the metrics reported highlighted the necessity for established standardized outcome measures to better compare and synthesize future research outcomes.

### 2.10. Risk of Biased Assessment

**Figure 2:**
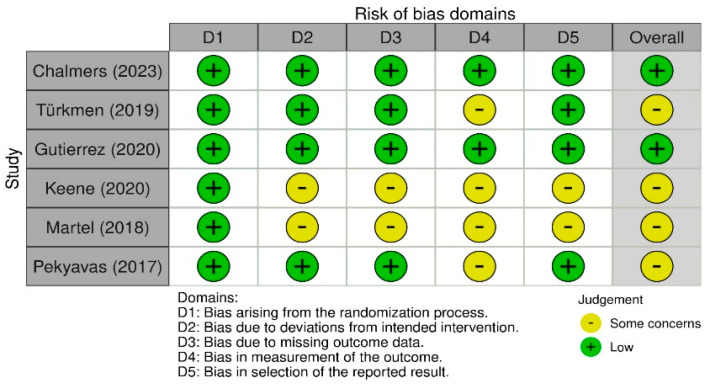
Rob 2 version of the Cochrane risk-of-bias tool for randomized control trials [29].

**Figure 3:**
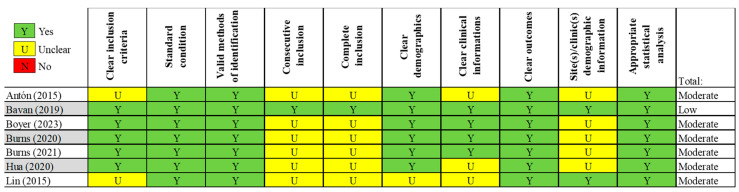
Joanna Briggs Institute (JBI) Critical Appraisal risk-of-bias tool for case series.

**Figure 4:**
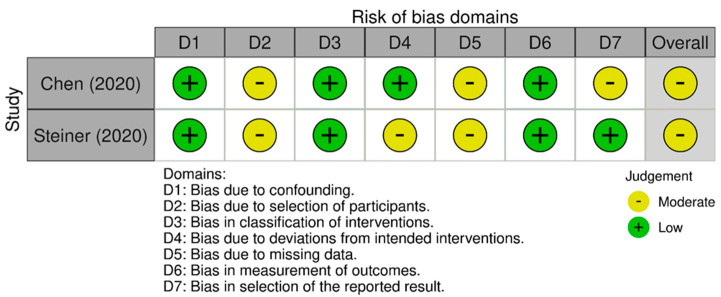
ROBINS-I risk-of-bias tool for case studies [30].

To assess the risk of bias in the included studies, three different tools were employed: the Rob 2 version of the Cochrane risk-of-bias tool for randomized control trials [29]; the Joanna Briggs Institute (JBI) Critical Appraisal tool for case series; and the ROBINS-I tool for non-randomized studies [30]. The RoB 2 tool is comprehensive, and structured into five key domains through which bias might be introduced into trial results (Figure 2). The JBI tool is a critical instrument for the evaluation of the methodological quality of studies, sorted into ten domains (Figure 3). ROBINS-I is a tool developed to assess the risk of bias in the results of non-randomized studies, using 7 domains (Figure 4). Two reviewers (G.N. and M.G.P.) worked independently to assess the risk of bias.

## 3. Results

### 3.1. Study Selection

A total of 463 articles were identified by the initial search, and 28 additional studies were identified through other sources. After the removal of duplicates, 481 articles were included in the analysis; out of these, 376 articles were excluded through the first title/abstract screening. Therefore, only 105 studies were considered for the full-text assessment, out of which only 15 studies fulfilled the inclusion criteria. Among the reasons for excluding articles, 46 were related to neurological pathology, 21 did not investigate the home environment, 10 articles involved patients without shoulder pain, 12 were excluded due to the use of exoskeleton or robotic devices, and 9 were inaccessible studies or did not involve shoulder joint (see Figure 1).

### 3.2. Study Characteristics

A total of 1453 patients were identified from the 15 studies included. Only 10 studies reported the percentage of female patients, adding up to 202 female patients, which accounts for 41.81% of the total population. The overall average age of the patients was 45.41 years ± 4.6 years old, with only 10 out of 15 articles providing this study characteristic (see Table 2). Regarding the variables reported, the most common input variables were as follows: Task executed (15 articles), Shoulder disease (15 articles), Monitoring system (14 articles), Age (12 articles), and Sex (10 articles). The most common output variable provided by the studies was the ROM (six articles) (see Table 3). The included studies recruited patients with various shoulder disorders. Specifically, eight studies enrolled patients being treated for rotator cuff (RC) pathology [21,31,32,33,34,35,36,37]; one study included patients with osteoarthritis and inflammatory conditions [34]; two studies involved patients with adhesive capsulitis [38,39]; and one study focused on patients diagnosed with type 2 subacromial impingement syndrome (SAIS) [40]. The remaining studies did not specify the shoulder disease.

The selected articles presented the following levels of evidence: five level IV case series [21,31,39,41,42]; one level IV cohort study [33]; one level II prospective comparative study [2]; one level II prospective control trial [38]; one level II prospective cohort study [32]; and six level I randomized control trials [34,35,36,37,40,43].

### 3.3. Monitoring System

Regarding the sensory technology employed in the studies, eight articles used wearable sensors [21,31,32,33,36,38,39,42], and six opted for camera-based systems [2,34,35,40,41,43]. The most used sensors in home-settings were inertial measurement units (IMUs), which integrate accelerometers and gyroscopes [21,38,39]. The integration of magnetometers in these units results in magnetic and inertial measurement units (M-IMUs), which were employed in four studies [31,32,33,42]. The quantity of sensors employed in the previously mentioned studies range from a single sensor [21,31,32,33] to configurations involving two [39], three [38] and up to four sensors [42]. In four studies, sensor units were provided and then placed on the subjects using elastic straps [38,39,42] or an arm sleeve [31]; while in three studies, IMU or MIMU units integrated into smartwatches were employed [21,32,33]. Except for the studies employing inertial sensors integrated into smartwatches, thereby positioned on the wrist, common placement of sensor units included the upper arm [31,38,39,42] and the wrist [38,39]. Other anatomical sites were the sternum [38], and the forearm, hand, and the lateral aspect of the torso [42]. The methodologies employed for data transmission exhibited variability. Certain studies transmitted sensor data wirelessly via Bluetooth to mobile phones [31,38], while others chose to store the data internally before uploading them to cloud storage for subsequent analysis [32,33]. The wearable device developed by Chen et al. [38] included not only IMU sensors, but also a mobile app called Patient App used by the patients, and a mobile app called Doctor App used by qualified health care professionals for monitoring patients’ progress. Hua et al. connected each sensor to a battery and a Raspberry Pi, centered on the front of the abdomen, serving as a computing platform capable of receiving, storing, processing, and potentially analyzing the sensor data [42]. Gutiérrez-Espinoza et al. [36] conducted a single-blinded randomized controlled trial to investigate an exercise program based on electromyography (EMG) sensors. EMG, using a percentage of maximal voluntary isometric contraction, was employed as a pragmatic tool to guide postoperative rehabilitation progression by sorting activation levels as low, moderate, high, and very high.

The effectiveness of using the previously mentioned wearable sensors may depend on how patients wear them. Recent trends indicate the growing use of low-cost non-intrusive visual tracking devices, such as camera-based monitoring systems, in tele-rehabilitation systems [22,35,38,40]. Three studies recorded patients’ movements using the Kinect system [2,41,43]. The use of a single camera offers the advantage of a straightforward setup, as it only requires connecting the camera to a device or installing the program on the television [40,43]. In comparison with wearable devices that require physical placement on the subject, camera-based solutions offer a more convenient and user-friendly solution. Türkmen et al. [35] proved the effectiveness of a video-based rehabilitation program in improving shoulder ROM, alleviating pain, enhancing functionality, and improving quality of life.

The development of a tele-rehabilitation system requires the integration and development of a graphical user interface, which enables users to receive exercise instructions [2] and observe two avatars, one demonstrating the correct execution of exercises and one reflecting the user’s actual execution, enabling them to discern any differences and facilitating movement correction [2,41,43]. Based on their execution, users receive real-time visual and acoustic feedback [2,41,43]. Additionally, the interface provides information about the performance improvement and the ongoing therapy session [2,43]. Therapists, on the other hand, can define new customized exercises for the users based on their performance [41]. The software allows the personalized adaptation of exercises and games, with the ability to adjust parameters such as speed, duration, precision, range of motion, number of repetitions, and difficulty levels [43].

In the realm of rehabilitation, virtual reality (VR) systems have been increasingly employed to further involve patients in their therapy [40,43]. Pekyavas et al. demonstrated the effectiveness of a VR exergaming program, such as the Nintendo Wii, as a valuable approach for patients undergoing rehabilitation.

### 3.4. Artificial Intelligence

The application of artificial intelligence (AI) and machine learning (ML) models in rehabilitation has become increasingly important for different purposes, such as classification, prediction, and the development of personalized treatment plans, as well as the enhancement of diagnostic accuracy [44,45,46,47,48]. These not only enhance treatment effectiveness but also facilitate more efficient and cost-effective care. One of the primary features of the system entails the incorporation of algorithms for recognizing patient movements, leveraging data gathered by the monitoring system [49]. Antón et al. [41] employed the Kinect system, incorporating an AI model, to enhance activity recognition. This system directly obtains the skeletal structure, encompassing 20 distinct joints. The initial detection of the body’s skeletal joints is pivotal in accurately detecting and analyzing joint movements, thereby enabling a comprehensive assessment of the quality of exercises performed by the patients. Built-in cameras, such as those in smartphones and tablets, along with AI, are now significantly revolutionizing human pose detection.

Following data acquisition, a ML algorithm was developed to undertake tasks such as classification, prediction, and treatment planning [50]. Traditionally, ML algorithms consists in two steps, i.e., feature extraction and pattern classification [21,31,42]. The segmentation of signals is necessary for the subsequent extraction of features. Differing from conventional fixed-size sliding window techniques [21,32,33], Bavan et al. [31] defines the boundaries of each movement by identifying time points where the velocity was closest to zero (see Table 4). This approach ensures the detection of out-of-distribution (OOD) data, i.e., eliminating non-exercise data [21]. Subsequently, from the acquired segmented signals, various features are computed, including mean, standard deviation, variance, kurtosis, range, and root mean square [21,31]. Alternatively, Hua et al. used the flattened structure of the data or the ROM as features sets [42]. For classification of exercises, the most commonly employed supervised ML models include the decision tree (DT) [31], the support vector machine (SVM) [31,42], the k-nearest neighbor (k-NN), and the random forest (RF) [21,31,42].

In recent years, deep learning (DL) techniques have shown outstanding performance in pattern recognition applications [21,51]. DL methods have been reported for the classification of various shoulder exercises, using either time series signals acquired from sensor data [21,32,33,39,42] or images captured by cameras. A neural network (NN) with a single hidden layer was implemented by Lin et al. [39]. Specifically, the NN consists of five neurons in the hidden layer, and six neurons in the output layer. The training algorithm employed in this NN architecture was the Back Propagation (Back Propagation Neural Network, BPNN), involving the iterative adjustment of the weights to minimize the error between the predicted and actual outputs. The implementation of multiple hidden layers resulted in the MultiLayer Perceptron (MLP) used by [42]. The combination of Convolutional Neural Networks (CNNs) and Recurrent Neural Networks (RNN) determines the Convolutional Recurrent Neural Network (CRNN) used in [33]. Incorporated into the Smart Physiotherapy Activity Recognition System (SPARS), the CRNN model takes the fixed-length windows of sensor data as input and is able to classify physiotherapy exercises and evaluate adherence to rehabilitation programs conducted at home. Two studies implemented the Fully Convolutional Neural Network (FCN) classifier to detect and classify physiotherapy exercises from the collected data [21,32]. The raw data acquired were processed using an overlapping sliding window segmentation with a ten-second window length to yield fixed-length input to the FCN classifier. The FCN model core proposed by [32] consists of 1D convolutional layers with rectified linear unit (ReLU) activation and batch normalization. Burns et al. implement the FCN classifier both for the binary classification task of differentiating physiotherapy activities from rest and activities of daily living (such as walking, working at a computer, etc.), as well as for the multiclass problem of discriminating between individual types of physiotherapy exercises [31]. A different approach was employed by [41] implementing the Dynamic Time Warping (DTW) algorithm as an exercise recognition method. Generally, the DTW algorithm assesses the similarity between two temporal time series. Specifically, Antón et al. adopted a variant of the DTW algorithm to compute the distances among trajectories for each limb to assess the correctness of the exercises’ execution. This involved a comparison between the trajectory path executed by the user with the corresponding stored trajectory [41].

To ensure robust model performance and achieve generalization to unseen data, the cross-validation method was employed during the training of ML models, providing a systematic approach to evaluate their predictive capabilities across diverse subsets of the dataset [21,31,39]. Different numbers of folds, usually 5 [21] or 10 [31], were used. Bavan et al. also implemented the Leave-one-subject-out validation (LOSOV) method to evaluate an algorithm’s performance [31]. For both traditional ML and DL approaches, model performance was evaluated using different metrics. Confusion matrices (CM) provide a tabular representation of classifiers’ performance [31,41,42]. Different metrics can be derived from the CM and used to assess the classification performances of the models, including accuracy [21,31,32,33,41,42], F1-score [21,32,33,42], precision or positive predictive value [21,31,32,33,42], sensitivity or recall or true-positive rate [21,31,32,33,42], specificity or true-negative rate [21,31,32]. Hua et al. also used speed and support, defined as the number of trials predicted for each label, to assess classifier performances [42]. Moreover, the Receiver Operating Characteristic (ROC) curve provides a graphical representation of the classification performance by illustrating the relationship between the false-positive rate and the sensitivity. It has been demonstrated that the area under the ROC curve (AUROC) is an excellent indicator of the classification performance because it visualizes classifier performance as a curve rather than a single scalar number, which conveys more information than many scoring measures [21,32].

### 3.5. Exercises Protocol

In the context of home-based rehabilitation exercises, there is a significant variability in the set of shoulder rehabilitation exercises for the patients (see Table 5).

The variability in the assigned protocol encompasses different domains, including the monitored exercises, the number of repetitions, as well as the number of sessions. Some protocols incorporated strengthening and stretching exercises to enhance the patient’s muscle strength and flexibility, and prevent stiffness [35,43]. The most used exercise, in the majority of studies, involved the flexion/extension movement in the sagittal plane [2,21,31,32,34,35,36,37,38,40,41,42,43], followed by abduction/adduction movement in the frontal plane [2,21,31,32,34,35,37,38,41,42,43], and the lateral/medial rotation [2,21,31,32,34,35,36,37,38,41,42]. The task of elevating the upper limb in the scapular plane was evaluated by three studies [32,35,39]. Other exercises aimed at addressing issues related to scapular mobility and stability include isometric scapular depression [36], scapular retraction [2,35], and scapular elevation [2]. Three studies also comprised external rotation with the arm abducted at 90° and horizontal abduction [2,32,42].

Additional shoulder rehabilitation exercises that were used in the selected studies included arm circumduction movement, wall slide [2,31], wall press [21,31,32], finger wall-climbing exercise [39], resisted row [21,32,42], pendulum exercise [38,39], resisted lat pull-down [21,32], push up [21,32,35], towel exercise [39], spiral rotation exercise in four steps [39], and back-shoulder circling exercise [39]. Pekyavas et al. also incorporated games such as boxing, bowing, and tennis [40]. Tasks related to ADLs were also evaluated, such as hand-to-mouth and hand-to-head [41] or hand-to-pocket [2]. Most exercises were carried out with the patient standing, while some exercises were also conducted in a seated [32] or a lying position [32,35].

### 3.6. Parameters Monitored during Exercises

The primary objective of the shoulder rehabilitation programs is to restore shoulder functionality, regain ROM, and enable most, if not all, of the ADLs. Studies primarily assessed shoulder ROM to evaluate rehabilitation progress and improvements in mobility and functional abilities. Starting from the three-dimensional (3D) coordinates of the 20 human joints, Antón et al. calculated the angle between the joints, and the angles between two limbs connected by a joint [41]. Particularly, they calculated the angles by the different parts of the body projected in the frontal plane (XY) and in the lateral plane (XZ). The raw data from each sensor were converted into orientation data, represented in either quaternion [31,38,42] or Euler Angles [31,42] representations, in order to acquire information regarding the 3D motion of the shoulder structure. Other studies monitored ROM at distinct follow-ups, providing insights into the progression of recovery over time [2,33,34,36]. Usually, these evaluations were conducted at baseline (preoperatively) [33,34,35,36,38], after 6 weeks [34,35,36], after 3 months [34,38], after 12 weeks [36], after 12 months [33], after 24 months [34]. Given the complexity of shoulder movements, shoulder ROM is typically computed for flexion and extension, adduction and abduction, and internal and external rotation [2,35,38].

Additional parameters not related to the shoulder joint, encompassing both physiological and biomechanical aspects, can be measured to gain a comprehensive insight into the patient’s response to rehabilitation exercises. Burns et al. collected both inertial data and heart rate data during physiotherapy exercise, but they did not use this physiological data as input to the AI model for a more comprehensive assessment of the patients’ performance [33]. The success of a rehabilitation program also depends on the daily behavior of the subjects. Gutiérrez-Espinoza et al., using the Xiaomi MI Band 3 Smart Bracelet, gathered data on daily movement behavior (DMB), encompassing information about physical activity (steps per day), sedentary behaviors, and sleep duration [36].

Moreover, validated clinical outcome measures were collected through questionnaires, facilitating the assessment of relevant metrics at baseline [2,32,33,34,35,36,38,40,43], after 4 weeks [32], 6 weeks [34,35,36,40], 1 month [38,40], 2 months [38], 8 weeks [32,37], 3 months [2,34,38], 6 months [2,37], 12 weeks [32,36,43], 12 months [33,37], and 24 months [34] of the rehabilitation program. The Visual Analog Scale (VAS) was employed to quantify pain levels (felt at rest, during activity, and at night) [34,35,36,38,40] and the level of function [34]. For the VAS scale, subjects indicated their perceived pain/function levels on a scale ranging from 0 (no pain or normal function) to 10 (worst pain or impaired function). Another scale used for pain assessment was the Numeric Pain Rating Scale (NPRS) [32,33].

Gutiérrez-Espinoza et al. evaluated shoulder function using the Constant–Murley questionnaire, which includes sub-scales addressing various dimensions such as pain, activities of daily living, and physical examination components related to active and muscular strength measures [36]. Three studies assessed pain and disability associated with shoulder pathology using the Shoulder Pain and Disability Index (SPADI) questionnaire [2,37,40]. To evaluate patients’ functional status, the Disabilities of the Arm, Shoulder and Hand (DASH) questionnaire [32,33,35,36,38], and the American Shoulder and Elbow Surgeons Standardized Shoulder Assessment Form (ASES) shoulder evaluation form [34] were used. The Western Ontario Osteoarthritis Score (WOOS) is a patient-reported outcome measure specifically designed specifically for assessing outcomes related to shoulder osteoarthritis [34]. The health-related quality of life (HRQoL) was assessed using the Short Form 12 (SF-12) [35] or the EQ-5D-5L (EuroQol-5 Dimensions, 5 Levels) score [37]. The Global Rating of Change (GROC) scale was used to gauge the overall levels of patients’ satisfaction [35]. This assessment was performed with a five-point Likert scale, where high scores are positively correlated with satisfaction. This scale provides a way for subjects to express their own perception of performance after the treatment. Other questionnaires were provided to patients covering other dimensions [2,33,37,43].

Adherence to the home-based rehabilitation program is crucial for maximizing treatment efficacy. Consistent engagement with the rehabilitation exercises enhance the likelihood of achieving therapeutic goals and improving overall functional outcomes. Adherence diaries, in which patients record their independent exercises, represent the most employed measure to assess adherence to home-based rehabilitation [33,37]. Two studies assessed adherence to the home-based program by computing the exercise completion rate, which represents the proportion of completed exercise sessions out of the total prescribed session [38,43]. Instead, Burns et al. provided the two-item Pain Self-Efficacy Questionnaire (patient self-efficacy) and the Patient Expectation Questionnaire score to explore potential predictors of physiotherapy adherence [32]. With respect to the acceptability of exercises, Martel et al. documented the perceived level of difficulty (PLD) and personal level of enjoyment (PLE) using a four-level analog scale at the conclusion of each session in the participant logbook [43]. This evaluation was performed using the Physical Activity Enjoyment Scale.

### 3.7. Quality Assessment

Regarding the risk-of-bias assessment in randomized trials, the studies conducted by Chalmers et al. [34] and Gutierrez et al. [36] demonstrated a low risk of bias. The remaining studies revealed certain concerns in specific domains, such as deviations from the intended intervention and the absence of outcome data [37,43]. In the case of non-randomized studies, the risk of bias ranged from low to moderate, with concerns regarding the selection of participants, deviations from intended interventions, and the prevalence of missing data [2,38]. Among these studies, the case series conducted by Bavan et al. stood out for having a low risk of bias [31]. The case series studies, which predominantly suggested a low risk of bias, were characterized by several unclear judgments, potentially indicating the need for more detailed information or further clarification [21,32,33,39,41,42]. The quality assessment suggested that, although many studies included in the systematic review exhibit high quality, there are specific areas where the risk of bias is elevated. These aspects should be considered when interpreting the results of the review.

## 4. Discussion

Traditional rehabilitation methods require regular visits to physiotherapy centers, entailing both cost and logistic challenges for many patients. The emerging field of remote patient monitoring shows promising advantages for enhancing patient outcomes in orthopedic care. The current trend is to leverage technology to enable efficient and effective treatment outside traditional clinical settings. Advances in technology and the latest AI models have facilitated the widespread adoption of home-based devices, providing an accessible and cost-effective platform for rehabilitation services.

This systematic review has provided insights into the evolving landscapes of systems designed for the remote monitoring of shoulder rehabilitation sessions conducted at home. The selection of the monitoring devices should prioritize usability and patient comfort. Wearable motion sensors, such as IMUs or M-IMUs, offer a non-intrusive solution for continuous monitoring of shoulder movements during rehabilitation exercises [16,25,26,27,28,29,30,32]. These devices are small, lightweight, non-invasive electronic devices, enabling real-time feedback and data collection, empowering patients to track their progress and adherence to prescribed exercises. These devices offer a non-intrusive and accessible means to capture motion data, allowing detailed assessments of exercise performance. The effectiveness of using these wearable sensors may depend on how patients wear them, highlighting the importance of proper positioning and adherence to wearing protocols for accurate data collection.

Camera-based monitoring systems, such as the Kinect system, have emerged as alternative solutions with the potential of offering a more user-friendly experience [2,17,24,31,34,35]. RGB and RGB-D cameras are the most prevalent types of cameras employed in such systems. However, these sensors are associated with certain limitations. For instance, the visibility of subjects is inherently dependent on the presence of adequate lighting conditions. In the realm of rehabilitation, virtual reality (VR) systems have been increasingly employed to further involve patients in their therapy [34,35]. The incorporation of serious games, challenges, and rewards into home-based rehabilitation programs offers an engaging and motivating platform for individuals undergoing therapy. This integration not only promotes active participation, but also facilitates progress tracking, holding the potential to transform therapy into a more enjoyable and interactive experience, ultimately enhancing patient engagement and treatment outcomes [16,25,26,27,28,30].

The heterogeneity among studies is not only related to the type of monitoring systems and AI models but also extends to the executed shoulder exercises and the monitored parameters. The primary objective of rehabilitation programs is to restore shoulder functionality, regain the range of motion (ROM), and enable most activities of daily living (ADLs). Consequently, a predominant focus in most studies involved the assessment of shoulder ROM, providing valuable metrics to evaluate improvements in shoulder mobility and the potential restoration of functional capabilities for everyday activities.

However, challenges and considerations arise in the usage of these monitoring systems. Factors such as patient adherence, detection of compensatory movements, and pain levels, should be addressed in the design and implementation of these technologies. Currently, the usage of a diary or questionnaires is commonly employed to evaluate adherence to home-based rehabilitation. In traditional rehabilitation sessions, therapists evaluate the patient’s performance during exercises and make necessary adjustments to optimize rehabilitation outcomes. This is more challenging in the home-based context. When assessing the execution of an exercise, various physiological parameters should be measured, including the respiratory frequency, or breathing rate, the heart rate, the body temperature, and others. This contributes to a holistic assessment of the patient’s overall condition. This is particularly important in evaluating the level of effort exerted during exercises or the level of pain, as traditional rehabilitation processes rely on therapist judgment, which may not be directly applicable in a remote setting. Out of all the studies analyzed in this review, only one study monitored heart rate, and another one monitored DMB.

### Limitations

This systematic review, while comprehensive in its approach, encountered various limitations. Firstly, the heterogeneity of the included studies poses a challenge. The variations in monitoring systems, artificial intelligence models, rehabilitation exercises, and monitored parameters hindered the ability to synthesize and compare results uniformly. This diversity, although reflective of the field’s richness, limits the ability to formulate generalized conclusions, suggesting the need for future research to use more rigorous methodological standards.

The technological aspect of remote monitoring raised concerns about user-friendliness, data privacy, and security. These factors play a crucial role in the widespread adoption and success of home-based rehabilitation programs but are not thoroughly addressed in the current literature.

These limitations should be carefully considered when interpreting the results of this review and in the design of future studies in this domain.

## 5. Conclusions

In conclusion, this review supports the integration of home-based monitoring devices alongside traditional rehabilitation methods, particularly crucial for patients with limited access to clinic-based therapy. This approach has the potential to facilitate a broader access to high-quality care and potentially reduce healthcare costs associated with in-person therapy. In designing and developing home-based monitoring devices, different key considerations must be addressed to ensure their effectiveness and widespread adoption. These systems must accurately measure and track patient progress, as well as provide reliable feedback and guidance to support the rehabilitation process. Scalability is also crucial to accommodate the specific needs of different individual users. Designing systems that are scalable allows for future updates and integration with emerging technologies, ensuring long-term relevance and utility. User experience is another critical aspect to be considered in the design of a home-based rehabilitation system. Ensuring ease of use, accessibility, and a positive overall user experience is crucial for widespread adoption, and creating an intuitive and user-friendly interface ensures easy engagement during rehabilitation sessions. Finally, potential ethical considerations need to be considered in the design and development of home-based rehabilitation systems. These concerns may include issues such as data privacy and security, informed consent, and the potential for misuse or abuse of these technologies. To address these concerns, measures such as implementing secure data storage and transmission protocols, establishing clear informed consent procedures, and incorporating safeguards against misuse or abuse should be integrated into the design of these systems [37].

While promising, these findings should be interpreted considering the methodological limitations and biases present in the available literature [38]. For future research, there is a clear need for standardization in the evaluation of remote rehabilitation devices to facilitate comparability across studies. Longitudinal research is also necessary to assess the long-term efficacy and safety of these technologies [39]. Additionally, cost-effectiveness analyses would be beneficial to justify their inclusion in healthcare systems [40]. It is incumbent, upon future research, to build on these findings, address the identified gaps, and corroborate the long-term benefits of these innovative healthcare solutions.

## Figures and Tables

**Table 1:** Search parameters in the databases.

Groups	Search Parameters
Medical application	“rehabilitation” OR “physiotherapy” OR “physiotherapy exercise *” OR “physical therapy” OR “rehabilitation exercise *” OR “telerehabilitation” OR “tele-rehabilitation” OR “tele rehabilitation” OR “tele-monitoring” OR “remote monitoring” OR “patient monitoring” OR “home-based”
Technology and kinematic/physiological data	“smartwatch” OR “motion tracking system *” OR “motion capture” OR “mocap” OR “contactless” OR “markerless” OR “camera-based” OR “camera *” OR “depth camera” OR “RGB-D” OR “RGBD” OR “RGB” OR “video” OR “video-based” OR “Kinect” OR “marker-less” OR “markerless” OR “wearable” OR “wearable sensor” OR “inertial sensor” OR “inertial measurement unit” OR “IMU” OR “MIMU” OR “accelerometer” OR “acceleration” OR “gyroscope” OR “IMU-based” OR “electromyography” OR “EMG” OR “surface electromyography” OR “surface electromyogram” OR “sEMG” OR “body-worn sensor” OR “skeletonization” OR “stress” OR “heart rate variability *” OR “hrv”
Artificial intelligence	“classification” OR “recognition” OR “pattern recognition” OR “unsupervised” OR “supervised” OR “deep learning” OR “spectrogram” OR “neural network” OR “artificial neural network” OR “ANN” OR “machine learning” OR “ML” OR “AI” OR “artificial intelligence” OR “Convolutional Neural Network” OR “CNN” OR “transformer” OR “classifier” OR “YOLO” OR “decision tree” OR “DT” OR “random forest” OR “RF” OR “k-nearest neighbors” OR “kNN” OR “k-NN” OR “Naive Bayes” OR “NB” OR “support vector machine” OR “SVM” OR “support vector machine classifier” OR “SVC”
Body segment	“shoulder” OR “rotator-cuff” OR “rotator-cuff” OR “shoulder pain” OR “shoulder injur *” OR “shoulder surgery” OR “rotator cuff injury” OR “frozen shoulder” OR “shoulder impingement” OR “upper extremity” OR “adhesive capsulitis” OR “dislocation” OR “Tendinitis” OR “Bursitis” OR “Fractures” OR “Arthritis” OR “Arthrosis”

(*) was used in the databases at the root of the word to find multiple endings.

**Table 2:** Study Characteristics.

First Author, Year	Study Design	Level of Evidence	Sample Size	Mean Age (SD)	Female Patients %	Shoulder Disease
Antón, 2015 [41]	Case series	IV	15	66	-	-
Bavan, 2019 [31]	Case series	IV	20	58.7	60%	RC
Boyer, 2023 [21]	Case series	IV	42	45 (13)	64.30%	RC
Burns, 2020 [33]	Cohort study (case series)	IV	140	-	-	RC
Burns, 2021 [32]	Prospective cohort study (case series)	II	42	45 (13)	64%	RC
Chalmers, 2023 [34]	Randomized clinical trial	I	HEP group: 46 PT group: 43	HEP group: 71.5 (7.9) PT group: 69.1 (7.5)	HEP group: 63% PT group: 54%	Osteoarthritis, inflammatory conditions, or RCTA
Türkmen, 2019 [35]	Randomized Controlled Trial	I	30	50.60 (8.54)	33%	RC
Chen, 2020 [38]	Prospective Control trial (case control)	II	24	MSR group: 53 (6.2) HEP group: 56.1 (13.3)	MSR group: 42.9% HEP group: 28.6%	AC
Gutiérrez-Espinoza, 2020 [36]	Randomized Control Trial	I	118	-	-	RC
Hua, 2020 [42]	Case series	IV	50	21.9 (4.0)	40%	-
Keene, 2020 [37]	Randomized Control Trial	I	708	-	-	RC
Lin, 2015 [39]	Case series	IV	13	-	-	AC
Martel, 2018 [43]	Randomized Control Trial	I	48	HEP group: 74.9 (7.1) YMCA group: 72.9 (6.7) Control Group: 72.7 (6.5)	HEP group: 75% YMCA group: 63%Control Group: 75%	-
Pekyavas, 2017 [40]	Randomized Control Trial-Therapeutic study	I	30	HEP group: 40.6 (11.7) Wii group: 40.33 (13.2)	HEP group: 86.7% Wii group: 93.3%	SAIS (type 2)
Steiner, 2020 [2]	Prospective comparative study (case control)	II	84	-	-	-

HEP = Home-Exercise Program; PT = Physical Therapy; YMCA = supervised group community-based exercise program; SD = Standard Deviation; AC = Adhesive Capsulitis; RC = Rotator cuff; RCTA = Rotator Cuff Tear Arthropathy; SAIS = Subacromial Impingement Syndrome; (-) = data not available.

**Table 3:** Input and Output variables.

First Author, Year	Input Variable	Output Variable
Antón, 2015 [41]	Age; shoulder disease; rehabilitation duration	Movement recognition performance
Bavan, 2019 [31]	Sex; age; arm dominance	Movement recognition performance
Boyer, 2023 [21]	Sex; age; rotator cuff tear thickness	Movement recognition performance
Burns, 2020 [33]	Age; BMI; arm dominance; symptoms duration; mechanism of injury; rotator cuff tear thickness; operative procedures; comorbidities; smoking; alcohol; opioid and cannabinoid intake; physical activity level; education; marital status; job demands; socioeconomic status; social support; patient self-efficacy	Adherence
Burns, 2021 [32]	Sex; age; BMI; Baseline pain level; physical activity level; job demands; education; socioeconomical status; patient self-efficacy	Adherence; Dose-response between physiotherapy activity and recovery; Movement recognition performance
Chalmers, 2023 [34]	Sex; age; affected arm; hand dominance; BMI; work status; comorbidities; ethnicity; smoking	ROM; patient-reported outcomes.
Türkmen, 2019 [35]	Sex; age; affected arm	Movement recognition performance
Chen, 2020 [38]	Sex; age; education	Adherence; Movement recognition performance
Gutiérrez-Espinoza, 2020 [36]	Age; BMI; dominant shoulder; duration of symptoms; socioeconomical status; occupation; education; previous treatment	Functional improvement; pain relief; ROM
Hua, 2020 [42]	Sex; age; BMI; History of upper extremity injury; physical abilities; health condition	Movement recognition performance
Keene, 2020 [37]	Age; rotator cuff disorder	Functional improvement; pain relief; adherence
Lin, 2015 [39]	Shoulder disease	Movement recognition performance
Martel, 2018 [43]	Sex; age; BMI	Functional capacities, cognitive function, health status, adherence, and acceptability
Pekyavas, 2017 [40]	Sex; age; diagnosis of type 2 SAIS and scapular dyskinesis	Efficacy of home exercise program and virtual reality exergaming; shoulder pain
Steiner, 2020 [2]	Age; shoulder complaints; ability to perform exercises without health risk; BMI	Efficacy

BMI = Body Mass Index; ML = Machine Learning; ROM: Range of motion.

**Table 4:** Artificial Intelligence Model.

First Author, Year	AI Model	Metrics	Cross-Validation Technique
Antón, 2015 [41]	DTW	CM; Accuracy	
Bavan, 2019 [31]	DT SVM k-NN RF	CM; Accuracy; Sensitivity; Precision; Specificity	10 folds CV; LOSOV
Boyer, 2023 [21]	k-NN FCN RF	Accuracy; Sensitivity; Specificity; AUROC; F1 score	5 folds CV
Burns, 2020 [33]	CRNN	Accuracy; Precision; Sensitivity; F1 score	-
Burns, 2021 [32]	FCN	Accuracy; Sensitivity; Specificity; AUROC; F1 score	-
Chalmers, 2023 [34]	-	-	-
Türkmen, 2019 [35]	-	-	-
Chen, 2020 [38]	-	-	-
Gutiérrez-Espinoza, 2020 [36]	-	-	-
Hua, 2020 [42]	RF (300 trees) LinearSVC k-NN MLP	CM; Accuracy; Precision; Sensitivity; F1 score; Speed; Support	-
Keene, 2020 [37]	-	-	-
Lin, 2015 [39]	BPNN	Accuracy	-
Martel, 2018 [43]	-	-	-
Pekyavas, 2017 [40]	-	-	-
Steiner, 2020 [2]	-	-	-

DTW = Dynamic Time Warping; DT = Decision tree; SVM = Support Vector Machine; SVC = Support Vector Classification; k-NN = k-Nearest Neighbor; RF = Random Forest; CRNN = Convolutional Recurrent Neural Network; FCN = Fully Convolutional Neural Network; MLP = Multilayer Perceptron; BPNN = Back Propagation Neural Network; CM = Confusion Matrix; AUROC = Area Under the Receiver Operating Characteristic; CV = Cross-Validation; LOSOV = Leave-One-Out Cross-Validation; (-) = data not available.

**Table 5:** Monitoring systems and Exercise protocol.

First Author, Year	Monitoring System (Type and Brand)	Number, Placement, and Wearability of Sensors	Task Executed		Recognition of Movement
			Shoulder Rehabilitation Exercise	Number, Repetitions, Protocol	
Antón, 2015 [41]	Camera (Microsoft Kinect system)	-	Shoulder abduction; Hands to mouth; Shoulder extension; Shoulder flexion; Hands to head; Shoulder rotation	*N* = 6	✓
Bavan, 2019 [31]	M-IMU (MetaMotion R)	N = 1; Upper arm (above elbow); Arm sleeve.	Shoulder abduction; Shoulder flexion; Wall slide; Wall press; Shoulder rotation	*N* = 5; Rept = 10	✓
Boyer, 2023 [21]	IMU	N = 1; Wrist; Smartwatches (Huawei Watch 2 smartwatches)	8 motions: Flexion; Abduction; ER; IR; Row; Elbow extension; Pull-down; Press-up6 simple motions: Elevation; Rotation; Row; Elbow flexion; Pull-down; Press-up	*N* = 18	✓
Burns, 2020 [33]	M-IMU	N = 1; Wrist; Smartwatches (Huawei Watch 2 smartwatches)	-	-	✓
Burns, 2021 [32]	M-IMU	N = 1; Wrist; Smartwatches (Huawei Watch 2 smartwatches)	9 motion types: Flexion; ER; IR; Press-up; Pull-down; Row; Abduction; Elbow flexion; Extension	*N* = 19; P = Patients were asked to complete their assigned exercises each day that they were not attending in-person physiotherapy.	✓
Chalmers, 2023 [34]	Camera	-	Active abduction; Active forward elevation; Active Internal rotation in adduction; Active External rotation in adduction	*N* = 4	-
Türkmen, 2019 [35]	Camera	-	Examples: Shoulder flexion with a stick; Scapular retraction; External rotation; Scapular adduction	P = Every day 3 sessions of 10 repetitions each session	-
Chen, 2020 [38]	IMU (BoostFix, COMPAL Electronics Inc).	N = 3; Sternum, upper arm, and dorsal wrist. Elastic straps	Shoulder Pendulum Exercise; Forward wall walking stretch; Lateral wall walking stretch; Cane stretch for shoulder flexion; Cane stretch for shoulder abduction; Cane stretch for shoulder external rotation; Cane stretch for shoulder internal rotation; Cane stretch for shoulder extension.	*N* = 8 P = Daily, 10 times each exercise, hold for 10 sec each exercise	-
Gutiérrez-Espinoza, 2020 [36]	EMG	-	Isometric Scapular Depression; Isometric Scapular orientation; External Rotation; Passive Flexion	-	-
Hua, 2020 [42]	M-IMU (Adafruit BNO055)	N = 4; Torso, upper arm, forearm, and hand; Elastic straps	Standing row; External rotation with arm abducted 90°; External rotation; Bicep curl; Forearm pronation/supination; Wrist curl; Lateral arm raise; Front arm raise; Horizontal Abduction	*N* = 9. Rept = 10	✓
Keene, 2020 [37]	-	-	External rotation; Flexion; Abduction of the Shoulder	*N* = 22	-
Lin, 2015 [39]	IMU	N = 2; Upper arm, and wrist; Elastic straps	Scapula exercise; Codman’s pendulum exercise; Finger wall-climbing exercise; Back-shoulder circling exercise; Towel exercise; Spiral rotation exercise in four steps	*N* = 6 Rept = 60 s	✓
Martel, 2018 [43]	Camera (Microsoft Kinect system)	-	Butt kicks, high knees, lateral launches, side steps;squats; leg extension; lateral shifting; balance; shoulder abduction/adduction; horizontal flexion and extension	P = Each exercise session lasted 55 min and included different exercises	✓
Pekyavas, 2017 [40]	Nintendo Wii	-	Posterior, anterior, and inferior capsule stretching; pectoral muscle stretching; serratus anterior muscle strengthening; bilateral shoulder elevation, and scapular mobility exercises. Bilateral shoulder elevation, boxing, bowling, and tennis games.	P = exercise program for 6 weeks, 2 days a week, and 45 min per day	✓
Steiner, 2020 [2]	Camera (Microsoft Kinect system)	-	Examples: Shoulder abduction/adduction; Shoulder flexion/extension; Shoulder external/internal rotation; Shoulder external rotation/internal rotation at 90° abduction.	*N* = 10 Rept = 5 days a week, 30 min per exercise, 6 months of training in total	✓

IMU = Inertial Measurement Unit; M-IMU = Magneto and Inertial Measurement Unit; EMG = Electromyography; ER = External Rotation; IR = Internal Rotation; *N* = number of exercises; Rept = repetitions; P = protocol; (✓) = implemented in the study; (-) = data not available.

## Data Availability

All data generated or analyzed during this study are included in this published article.
